# Estimation of the time course of neurotransmitter release at central synapses from the first latency of postsynaptic currents

**DOI:** 10.1016/j.jneumeth.2011.12.015

**Published:** 2012-03-30

**Authors:** Federico Minneci, Roby T. Kanichay, R. Angus Silver

**Affiliations:** Department of Neuroscience, Physiology and Pharmacology, University College London, Gower Street, London WC1E 6BT, UK

**Keywords:** Release time course, Vesicle, Synaptic transmission, First latencies, Binomial model

## Abstract

Measurement of the release time course (RTC) and of the quantal content is important for quantifying synaptic precision and understanding the molecular basis of the release process at central synapses. In theory, the RTC can be determined directly from the histogram of first latencies of quantal events only if a maximum of one vesicle is released per trial, but at most synapses multiple vesicles are released. Traditionally, first latency histograms have been corrected for multiple releases using a simple correction, derived by Barrett and Stevens (BS; 1972b) for quantifying release at the neuromuscular junction. This correction has also been used to quantify release at central synapses. We show, by combining an analytical approach and numerical simulations of stochastic quantal release, that the BS correction gives a biased estimate for RTC and quantal content. The bias increases with release probability, and is therefore particularly problematic for central synapses. We show that this is due to assuming infinite availability of releasable vesicles and we derive a formula for estimating the RTC from first latencies without this assumption. The resulting ‘binomial correction’ requires knowledge of the maximum number of quanta that can be released following an action potential (*N*), which can be estimated with variance-mean analysis. We show with simulations that estimating RTC and quantal content from first latencies using the binomial correction is robust in the presence of noise and when release probability is non-uniform. We also provide an alternative method for estimating RTC from the first latencies when *N* cannot be determined.

## Highlights

► Method for estimating vesicular release time course from PSC first latencies. ► Analytical derivation of binomial model of release at central synapses. ► Systematic tests of robustness with biologically realistic simulations. ► Generalization of existing first latency correction based on Poisson model.

## 1 Introduction

### 1.1 The time course of vesicular release

A fundamental determinant of the strength and temporal fidelity of transmission at a synaptic connection is the rate and time course of vesicular release. The release time course (RTC) defines the number of neurotransmitter vesicles (quanta) released per unit time from the presynaptic terminal in response to an action potential, and it is a determinant of the kinetics of the postsynaptic current (Van der Kloot, 1988; Diamond and Jahr, 1995; Isaacson and Walmsley, 1995; Geiger et al., 1997; Sargent et al., 2005). Since the kinetics of postsynaptic currents greatly influence spike time precision (Galarreta and Hestrin, 2001; Cathala et al., 2003), the fast RTCs observed at some synapses have been implicated in underlying spike time precision at these synaptic connections (Geiger et al., 1997; Jonas et al., 2004; Sargent et al., 2005; Taschenberger et al., 2005; Kanichay and Silver, 2008).

The time course of the vesicular release rate is also the ultimate and observable output of the molecular process underlying neurotransmitter release. Using the RTC as an assay of the release process has provided insight into the molecular mechanisms underlying vesicular release (Kerr et al., 2008; Bucurenciu et al., 2010), or what plastic changes they may undergo (Waldeck et al., 2000; Lin and Faber, 2002), and is a determinant of the information transmission capability they possess (Rieke et al., 1997). It is therefore important to develop methods to determine the kinetics of vesicular release accurately. Given the expansion in knowledge in this field and the refinement of available techniques, it is also increasingly important to improve tools that are used for such analysis (Stevens, 2003).

### 1.2 Methods for estimating the RTC

Deconvolution of the average evoked postsynaptic response with the uniquantal current yields the release rate function, provided quantal currents (QCs) are constant and add linearly (Van der Kloot, 1988; Diamond and Jahr, 1995; Chen and Regehr, 1999; Vorobieva et al., 1999; Schneggenburger and Neher, 2000; Hefft and Jonas, 2005; Sargent et al., 2005). However, this premise may not be fulfilled at many synapses. Postsynaptic receptor saturation and desensitization due to multivesicular release (Silver et al., 1996; Wadiche and Jahr, 2001; Foster et al., 2002) or delayed clearance and neurotransmitter spillover can cause non-linear interaction between quanta (DiGregorio et al., 2002; Taschenberger et al., 2005). More recent studies have accounted for non-linearity in the postsynaptic response (Neher and Sakaba, 2001; Scheuss et al., 2007), but the analysis is complicated and may not be applicable to all synaptic connections.

The release rate can also be directly deduced from the latency distribution of quantal events, which can be constructed by measuring the latency of individual quanta from recordings of postsynaptic events (Barrett and Stevens, 1972b; Isaacson and Walmsley, 1995; Geiger et al., 1997; Bennett and Kearns, 2000; Sargent et al., 2005). A limitation of this approach is that when multiple overlapping quantal responses occur, only the latency of the earliest quantal event can be measured unambiguously, as variance in quantal size and the presence of noise in the recordings make it difficult to estimate the latency of quanta that do not rise directly from the baseline. The resulting distribution of first latencies of postsynaptic events neglects the occurrence of vesicles released at a later time point, and thus is biased towards quanta released early during the release process. To address this problem Stevens and colleagues (Stevens, 1968; Barrett and Stevens, 1972a, 1972b) developed a method that estimates the later occurring events and corrects the RTC derived from the first latencies of postsynaptic events accordingly. This correction was derived for and first applied to the neuromuscular junction (NMJ), where there are many releasable vesicles. The process was modelled by release of vesicles with replacement, implying an infinite availability of vesicles. This approach was used to study the RTC at the amphibian NMJ under conditions in which the number of releasable vesicles was large and the vesicular release probability was low (Barrett and Stevens, 1972b; Baldo et al., 1986). Later, the same approach and correction were applied to large auditory synapses in the central nervous system (Isaacson and Walmsley, 1995; Taschenberger et al., 2005) and various hippocampal synapses (Geiger et al., 1997; Kraushaar and Jonas, 2000; Kerr et al., 2008).

In this study we use mathematical analysis and simulations of synaptic release to assess the validity of the correction method proposed by Barrett and Stevens for central synapses, using a minimal model with few assumptions about the release. Moreover, we present a generally applicable analytical solution to the problem of obtaining RTC from the first latencies. This requires estimation of the number of readily releasable vesicles in order to produce an unbiased correction. Finally we outline, for cases when such estimation is impossible, a method for deducing reliable information about the RTC from the first latencies without the need of any correction.

## 2 Methods

Some of the analytical results were obtained using Mathematica 7.0 (Wolfram Research, Champaign, IL, USA). Analysis of curves and figure plotting were performed with custom-made software written in Igor Pro 6 (Wavemetrics, Lake Oswego, OR, USA), or using Neuromatic (http://www.neuromatic.thinkrandom.com) within the Igor Pro environment.

### 2.1 Functions used to model the RTC

For calculations that required an explicit form of release rates, the probability density function for the RTC was modelled with either a Gamma distribution or a Gaussian function. For the Gamma case, the RTC was modelled by a shifted Gamma distribution with the Gamma parameter *k* equal to 2, i.e. a function proportional to (*t* − *t*_0_) exp(−(*t* − *t*_0_)/*T*) where *t* is time, *T* is a constant and *t*_0_ is a horizontal offset (this type of function has previously been used for this purpose: see Barrett and Stevens, 1972b; Baldo et al., 1986). For the Gaussian case, a truncated Gaussian function with mean equal to 3 standard deviations was used. The function is equal to zero for values of time less than or equal to zero, which was always considered to be the time at which the action potential reached the presynaptic terminal. In both cases, the constants were adjusted so that the standard deviation of the RTC function was equal to 300 μs, as common in central synapses (Isaacson and Walmsley, 1995; Geiger et al., 1997; Kraushaar and Jonas, 2000; Schneggenburger and Neher, 2000; Taschenberger et al., 2005), except for some of the simulations in Fig. 8C and D (see Section 2.4.1 below). Unless explicitly mentioned, only results obtained with a Gamma distribution are reported in the paper.

### 2.2 Numerical simulations of synaptic activity

**Fig. 1:**
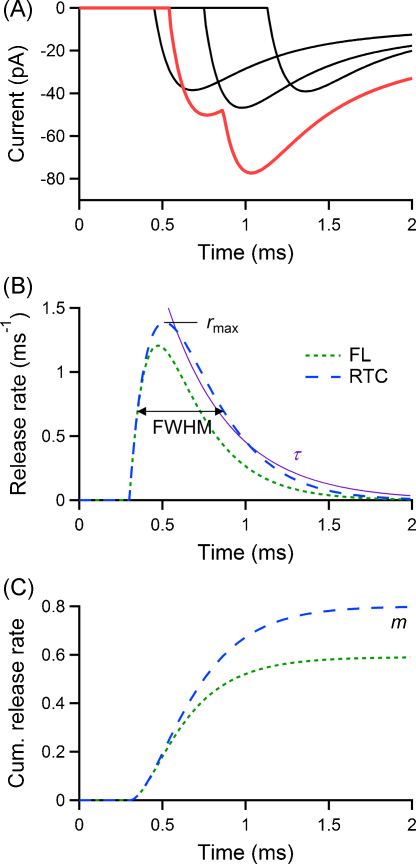
Multiquantal events mask the vesicular release time course. The problem is illustrated using a simulation of stochastic release at a synapse with four releasable vesicles, a vesicular release probability of 0.2, and RTC modelled by a shifted Gamma distribution with a standard deviation of 300 μs. For postsynaptic responses, single quantal events are modelled by a multi-exponential function with kinetics detailed in Section 2. (A) Sample EPSCs from the simulation (uniquantal events in black, one multiquantal EPSC in red). (B) Release rate functions for the first quantum (first latency; FL) and for all quanta (RTC), and indication of parameters used to quantify the RTC: maximal release rate (*r*_max_), full width half maximum of the curve (FWHM), decay time constant (*τ*). (C) Corresponding cumulative release rate curves, and indication of the quantal content (*m*), equal to the asymptotic value of the cumulative RTC curve. (For interpretation of the references to color in this figure legend, the reader is referred to the web version of the article.)

**Fig. 2:**
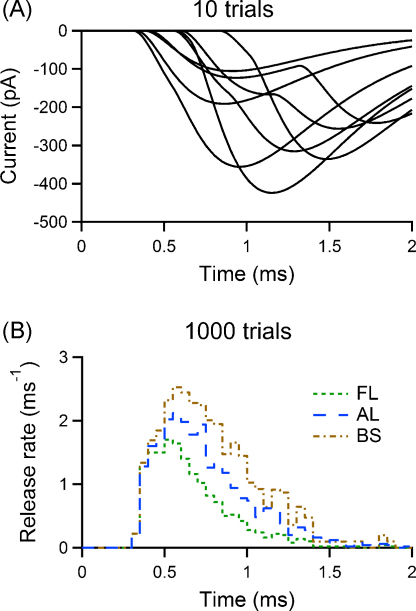
Numerical stochastic simulations reveal an error in the estimation of the release time course from the first latency distribution using the Barrett and Stevens formula. Results of the simulation of release at a synapse with three releasable vesicles, a failure probability of 0.2, no replacement of released vesicles during single trial, and RTC modelled by a shifted Gamma distribution with a standard deviation of 300 μs (these as well as the EPSC parameters are found at central synapses). Plots are truncated at *t* = 2 ms in order to allow a better visualisation of the latency time courses. (A) Ten selected sample postsynaptic traces (EPSCs and failures) from the simulation are shown in black. (B) Scaled histograms of the first latencies of postsynaptic responses (FL), the actual latencies of all quantal events (AL), and the FL histogram corrected with the Barrett and Stevens formula (BS) for a simulated experiment with 1000 trials (histogram bins of 50 μs).

All numerical simulations were performed with custom-made software written in Igor Pro that performs Monte Carlo simulations of synaptic transmission. For simulations that involved postsynaptic currents, existing software (Sargent et al., 2005; Saviane and Silver, 2006b) was extended. All synapses consisted of *N* releasable quanta. At each stimulation trial, a single action potential reached all synaptic release sites simultaneously. For each releasable quantum, a random number was picked in the interval [0,1) and compared to the assigned vesicular probability *P*: if the number was smaller than *P*, the quantum was released and contributed to the total current. In most simulations, *P* was constant for all quanta. In the case of non-uniform release probability (Section 3.3.3), a *P* value was picked for each releasable quantum from a truncated normal distribution (0 < *P* < 1) centred on the average value $\bar{P}$ and with a coefficient of variation equal to CV_*P*_; sets of *P* values were picked iteratively until they approximated the desired average and CV values with a precision of 1%. For each released quantum, a latency value was then picked from a normalised distribution with the same shape as the RTC. Quanta released on the same trial were summed linearly to produce simulated postsynaptic responses. Individual quantal currents were modelled by a waveform with kinetics and amplitudes representative of excitatory quantal currents recorded at central synapses. The function used was the following (Nielsen et al., 2004; modified from Bekkers and Stevens, 1996):

(1) $$QC(t)=A_{1}\cdot {\left(1-e^{-((t-L)/\tau_{R})}\right)}^{n}\cdot (A_{2}\cdot e^{-((t-L)/\tau_{D1})}+(1-A_{2})\cdot e^{-((t-L)/\tau_{D2})})$$

where *L* indicates the latency of the quantal event. The other parameters in Eq. (1) were constant for a particular synapse except for *A*_1_, which was allowed to vary in order to achieve a CV of the quantal peak amplitude equal to CV_*Q*_. For Fig. 1, the values for the constants were as follows: *A* = −100 pA, *τ*_*R*_ = 0.15 ms, *τ*_*D*1_ = 0.4 ms, *τ*_*D*2_ = 5 ms, *n* = 1, CV_*Q*_ = 0.3. For Figs. 2 and 5, all values were adjusted so that the resulting postsynaptic currents resembled those at hippocampal synapses (Geiger et al., 1997). For Fig. 7, values were as for Fig. 2 but *A*_1_ was decreased to obtain the required value of signal to noise ratio.

### 2.3 Labelling of curves in the figure legends

In all figures, the curves present in release rate plots were labelled as follows. The label for the analytical release rate function of all latencies is “RTC”, while in simulation figures the label for the actual latency curve (the scaled version of the histogram including all simulated latencies) is “AL”. The labels for other release rate curves are used consistently for both numerical simulations and analytical calculations: “FL” for the scaled simulated histogram or the analytical release rate function of first latencies, “BS” for those obtained by correcting FL with the BS formula in Eq. (14), and “Bin” for those obtained by correcting the relevant “FL” curve with the binomial formula in Eq. (13).

### 2.4 Details of the analysis

To average out stochastic variability, the analysis illustrated in Figs. 6B–D and 7E and F (see Sections 3.3.3 and 3.3.4) was performed on curves obtained from an average of 10 simulations of 2 × 10^7^ stimulation trials each.

In the analysis reported in Sections 3.3.1 and 3.3.4, the template match search was performed using Neuromatic, employing the algorithm described by Clements and Bekkers (1997). Briefly, a template was obtained by trimming the quantal postsynaptic response used in the simulation to 3 ms duration. The algorithm then slid the template along each trace, rescaling it vertically to find the best fit with the data. If a detection criterion, based on the optimum scaling factor and the quality of the fit, crossed a certain threshold, the trace was considered to contain a response and its latency obtained as the rising point of the template. The threshold for the detection criterion was kept at −3 throughout the analysis, as suggested by Clements and Bekkers (1997).

In the same analysis, the significance of the difference between pairs of curves was tested using the Kolmogorov–Smirnov test. When analysing binned data, this test gives an approximate *p*-value. We used this approach because the chi-square test, which is usually more appropriate for binned data, relies on the assumption of high numbers of counts in all bins, which is often not the case for latency histograms.

#### 2.4.1 Analysis of reliability and sensitivity

For the analysis described in Section 3.3.5, multiple simulations were run for each point in the (*N*, *P*) plots, and their outcomes were compared as described below. For each plot, stochastic simulations were run until the number of postsynaptic successes reached a specific value (200 successes for Fig. 8A and C, 500 successes for Fig. 8B and D). Simulations were restricted to cases that required less than 5000 stimulation trials, and had a failure rate higher than 10% (Fig. 8A and C) or 5% (Fig. 8B and D).

For the analysis of the reliability of the binomial correction (plots in Fig. 8A and B), 20 simulations with identical parameters were run multiple times for each point in the (*N*, *P*) plots. The RTC was modelled with a Gamma distribution with standard deviation of 300 μs. The actual latency curve (the scaled version of the histogram including all simulated latencies) was obtained from each of 10 simulations, while the binomial curve (the binomial corrected version of the scaled first latency histogram) was estimated for the remaining 10 simulations. The decay time constant (*τ*, defined in Section 3.1) was determined from the fit of an exponential function to each scaled histogram, and the two sets of 10 values were compared using the unpaired *t*-test with significance level set at 0.05. This was repeated 6000 times and the false positive rate (*α* value) was obtained and plotted for the corresponding (*N*, *P*) values.

For the analysis of the sensitivity of the binomial correction (plots in Fig. 8C and D), 20 simulations were run multiple times for each point in the (*N*, *P*) plots. The simulations had the same number of trials, *N* and *P*, but 10 used the original RTC with a standard deviation of 300 μs, while the RTC for the remaining 10 had an increased half-width. The horizontal offset of the RTC was always kept constant. The decay time constant was calculated for the binomial curve from each simulation, and the two sets of 10 values were compared using the unpaired *t*-test with significance level set at 0.05. This was repeated 2500 times so that the false negative rate (*β* value) was obtained. The whole procedure was repeated with the half-width of the second synapse increasing progressively (30 μs increments), until the power of the test (equal to 1 − *β*) reached the threshold value of 0.5. At that point the procedure was stopped and that that increase in half-width was plotted for the corresponding (*N*, *P*) values.

### 2.5 Derivation of the binomial correction

Here we derive our model for the simplest description of the synaptic release process with no replacement of released vesicles.

#### 2.5.1 Definitions, assumptions and basic quantities

In this basic model, we consider only the readily releasable pool (RRP) of vesicles at a synaptic connection, neglecting replenishment during the short time course (∼1 ms) of the release process (see Section 4.1.2). We assume that the RRP is composed of a finite number *N* of identical releasable units (vesicles), each of which can be released independently of the others at any stimulation (see Katz and Miledi, 1965; Isaacson and Walmsley, 1995). This definition includes many equivalent cases, from that of a single release site with *N* vesicles (multivesicular release), to the other extreme case of a synapse composed of *N* identical and independent release sites which can release a maximum of one vesicle per trial (univesicular release sites). In all cases, *N* is the total number of vesicles available for release by an action potential.

After the stimulation, each vesicle can be released at some point in time or remain unreleased. We define *r*_1_(*t*) as the release rate function for a single vesicle, and we assume *r*_1_(*t*) to be the same across vesicles. It is given by a scaled probability density function, such that the probability of being released in a particular interval of time is given by its integral over that interval. In our case, this function is equal to zero for *t* ≤ 0 (see Section 2.1). If *P* is the overall probability that the single vesicle will be actually released after one stimulation, *r*_1_(*t*) = *P* · *pdf*(*t*), so that $\int_{0}^{\infty }r_{1}(t)dt=P$, where the probability density function pdf(*t*) is normalised to 1. The probability of a vesicle being released up to time *t* is given by its integral function *c*_1_(*t*), defined as the cumulative release rate function for a single vesicle, which therefore has the property that $\underset{t\to +\infty }{\lim}c_{1}(t)=P$.

Other relevant functions are defined as follows:

*f*_1_(*t*) gives the cumulative probability of no release for a single vesicle, so that $\underset{t\to +\infty }{\lim}f_{1}(t)=1-P$;
*f*_*N*_(*t*) gives the cumulative probability of no release occurring for *N* vesicles (the whole synaptic connection), with $\underset{t\to +\infty }{\lim}f_{N}(t)=F$ if *F* is the proportion of failures for the whole synaptic connection;
*s*(*t*) and *S*(*t*) are the release rate and cumulative release rate functions for the first release of the whole synapse (“first latency” functions, as in Barrett and Stevens, 1972b), so that $\int_{0}^{\infty }s(t)dt=\underset{t\to +\infty }{\lim}S(t)=1-F$;
*r*_*N*_(*t*) is the release rate function for *N* vesicles (the whole synapse), i.e. the RTC;
*α*_*BS*_(*t*) is the function that Barrett and Stevens (1972b) use to estimate *r*_*N*_(*t*).

In summary, the only important assumptions in this model are:

• The release of one of *N* vesicles is independent of release of any other vesicle.
• *r*_1_(*t*) is uniform across vesicles.
• The underlying process of spontaneous release is negligible for our purposes.

Note that all definitions can be easily adapted to the point of view of a synapse composed of *N* univesicular release sites. For example, *r*_1_(*t*) becomes the release rate function for release from a single site. In what follows, the more general case of *N* independently releasable vesicles will be examined.

The RTC function of the whole synapse can be written as a function of the release rate function of the single vesicle as:

(2) $$r_{N}(t)=N\cdot r_{1}(t)$$

while the cumulative functions *c*_1_(*t*), *f*_1_(*t*) and *S*(*t*) are given by:

(3) $$c_{1}(t)=\int_{0}^{t}r_{1}(\tau )d\tau$$

(4) $$f_{1}(t)=1-c_{1}(t)=1-\int_{0}^{t}r_{1}(\tau )d\tau$$

(5) $$S(t)=\int_{0}^{t}s(\tau )d\tau$$

The probability of having no quantal events between time 0 and time *t* is:

(6) $$f_{N}(t)=f_{1}{\left(t\right)}^{N}={\left(1-c_{1}(t)\right)}^{N}$$

Rearranging and using Eqs. (2) and (3) we have:

(7) $$c_{1}(t)=1-f_{N}{\left(t\right)}^{1/N}$$

(8) $$r_{N}(t)=N\cdot \frac{d}{dt}(1-f_{N}{\left(t\right)}^{1/N})=-N\cdot \frac{d}{dt}(f_{N}{\left(t\right)}^{1/N})$$

Thus, measuring the failure probability as a function of time allows calculation of *c*_1_(*t*) when *N* is known, so that the RTC function *r*_*N*_(*t*) can be obtained. Also, expressing in terms of the first latency cumulative function *S*(*t*) gives:

(9) $$1-S(t)=f_{N}(t)=f_{1}{\left(t\right)}^{N}$$

(10) $$r_{N}(t)=-N\cdot \frac{d}{dt}({\left(1-S(t)\right)}^{1/N})$$

#### 2.5.2 Analytical comparison with the BS formula

Alternatively, an equivalent expression for *r*_*N*_(*t*) can be derived in a manner similar to that taken in Barrett and Stevens (1972b). With *N* releasable vesicles, the probability density of observing the first quantal event at some time *t* is the product of the probability of no prior event having occurred, times the probability density of an event occurring at *t*. However, for single vesicles, or for release sites that can release a maximum of one quantum per trial, release and non-release are not independent variables. If a given vesicle is released at time *t*, the probability that it was not released before *t* is by definition equal to 1. In order to have a first latency at time *t*, one vesicle must be released exactly at *t* (then, for that vesicle it is not necessary to impose that it was not released before *t*), and *N* − 1 vesicles must be released not before *t*. After *t*, these *N* − 1 vesicles can do anything: even if all of them are released at *t*, that does not matter for the first latency, which still happens at *t*. Moreover, any of the *N* identical vesicles can be the one which is released at *t*. The first latency release rate function is therefore:

(11) $$s(t)=f_{1}{\left(t\right)}^{N-1}\cdot N\cdot r_{1}(t)$$

Note that this can be derived analytically, simply using Eqs. (5), (9) and (4) in turn:

(12) $$s(t)=\frac{d}{dt}S(t)=\frac{d}{dt}(1-f_{1}{\left(t\right)}^{N})=f_{1}{\left(t\right)}^{N-1}\cdot N\cdot r_{1}(t)$$

Using Eqs. (2) and (9), this expression for *s*(*t*) gives the formula for *r*_*N*_(*t*):

(13) $$r_{N}(t)=\frac{s(t)}{{\left(1-S(t)\right)}^{\left((N-1)/N\right)}}$$

It is important to note that the assumption of no vesicle replacement is compatible with the description of our model as a simple binomial model of synaptic release, and that in this framework the BS model constitutes an approximation of ours in the Poisson limit, as detailed in Appendix A.

## 3 Results

### 3.1 The problem and the traditional solution

A direct way to determine the vesicular RTC is to measure the latencies of postsynaptic events, e.g. excitatory postsynaptic currents, evoked by the release of individual vesicles. However, in case of multivesicular release only the latency of the response to the first vesicle can usually be determined reliably. To illustrate this we simulated postsynaptic currents evoked by an action potential for a synaptic connection with four releasable vesicles, a vesicular release probability of 0.2, including quantal variability and a presynaptic jitter in vesicular release with standard deviation of 300 μs (Fig. 1; see Section 2). In Fig. 1A, three of the postsynaptic currents correspond to the release of a single vesicle, while on the remaining trial (red line) multiple vesicles were released. The latency distribution of the first postsynaptic events (the first latencies) ignores the release of all vesicles released after the first one in a particular trial. Consequently, a vesicular RTC deduced from the first latency distribution of postsynaptic events neglects the contribution of later vesicles. This is illustrated in Fig. 1B which shows the time course of vesicular release, as time-dependent release rate, derived from the latencies of the first quantal events (green line) and the actual RTC underlying the release process (blue line; both calculated analytically using the synaptic parameters mentioned in Fig. 1A). A discrepancy between the two curves is evident. To quantify the vesicular RTC, parameters such as the half-width (i.e. the full width at half maximum, FWHM), the decay time constant (*τ*, obtained from an exponential fit of the decreasing part of the RTC) and the maximal release rate (*r*_max_) can be used (Fig. 1B). Moreover, the integral of the release rate can be used to determine the quantal content (*m*) of the synaptic connection (Fig. 1C). For this example, the half-width of the RTC derived from first latencies is 426 μs compared to 519 μs for the actual release; similarly, the decay kinetics (*τ* of 277 μs vs 302 μs), the peak release rate (1.21 ms^−1^ vs 1.39 ms^−1^) and the quantal content (0.59 vs 0.8) are different. This emphasises the need to correct the estimate of the vesicular RTC derived from the first latencies, as it is biased towards release events occurring early during the release process.

#### 3.1.1 The Barrett and Stevens (BS) correction

In a seminal paper (Barrett and Stevens, 1972b; see Stevens, 1968 for a detailed derivation) a solution to the problem was proposed as follows. If *s*(*t*) is the rate of occurrence of the first release event at time *t* after a stimulation occurring at time 0, and *S*(*t*) is the corresponding cumulative rate (i.e. probability), then 1 − *S*(*t*) gives the probability that no release has occurred up to time *t*. Thus, if there is an infinite availability of quanta at each stimulation, it is true that *s*(*t*) = (1 − *S*(*t*)) · *α*_*BS*_(*t*) where *α*_*BS*_(*t*) is the rate of observing a quantal release at time *t* from the whole synapse. Such a function is then an estimate of the RTC of the whole synapse, and can be written explicitly as:

(14) $$\alpha_{BS}(t)=\frac{s(t)}{1-S(t)}$$

Measuring the first latency distribution gives an estimate of *s*(*t*), and measuring the failure probability as a function of time gives an estimate of *S*(*t*), so that an estimate of the RTC can be obtained from the experimental results. It is easy to see that the estimate is more reliable if the failure probability is high; otherwise, 1–*S*(*t*) becomes small at large times making the estimate noisy (with no failures, the estimation is impossible for times after the longest first latency). This correction implicitly assumes an infinite availability of quanta within each stimulation episode; in fact, it assumes that the function *α*_*BS*_(*t*) does not depend on the number of vesicles available for release following an action potential – that is, the RRP. However, the replenishment of the RRP after an action potential is much slower than the RTC (Saviane and Silver, 2006a; see Section 4.1.2). This means that Eq. (14) is not generally applicable. This becomes immediately apparent when considering the extreme case of a synaptic connection that has only one releasable vesicle. In this case, the first latencies describe the actual RTC with no need for correction. Using Eq. (14) to estimate the RTC would falsely assume overlapping vesicular release and thus incorrectly estimate the RTC.

Fig. 2 shows an example of simulation of the effect of the BS formula at a synapse with three releasable vesicles and failure probability of 20%. These quantal parameters were chosen to be in the range common to many small central synapses (Dobrunz and Stevens, 1997; Geiger et al., 1997; Kondo and Marty, 1998; Kraushaar and Jonas, 2000; Oertner et al., 2002). In this stochastic simulation of synaptic release, that includes quantal variability, no replacement of released vesicles can occur before the next quantum is released. Fig. 2A shows 10 sample postsynaptic currents, while the plot in Fig. 2B shows a histogram (50 μs bins) including all latencies (blue line), one including just first latencies (green line), and one obtained using the BS correction of the first latencies (brown line) for a simulated experiment with 1000 presynaptic stimulations. It is evident that the correction overestimates the RTC, and the error is comparable to that obtained by estimating the RTC with the first latency histogram itself.

### 3.2 A statistical description of synaptic release with no replacement of vesicles

In order to gain an unbiased estimate of the RTC and to quantify the bias of the BS correction analytically, we derived the relationship between the first latency curve and the RTC for a finite number of releasable vesicles.

#### 3.2.1 The binomial correction

To account for the decrease in the availability of quanta during the release process that follows an action potential, we used a model that describes release without replacement. Similar derivations are present in the literature but they either draw the same conclusions as in Barrett and Stevens (1972b), or use more complicated models with additional parameters (see Section 4.1). We derived the simplest description of the synaptic release process with no replacement of vesicles (see Section 2.5 and Appendix A), obtaining a simple binomial model of release. The RTC, which is equal to the release rate function for the whole synapse *r*_*N*_(*t*), can be estimated in our model using Eq. (13), where *s*(*t*) and *S*(*t*) are the same functions used in Section 3.1.1. Eq. (13) allows estimation of the RTC from experimental measurements of the first latencies of postsynaptic responses when *N* is known. Note that *r*_*N*_(*t*) = *s*(*t*) when *N* = 1. This formula shares with Eq. (14) the limitation that, as the cumulative first latency distribution tends to 1, extracting information about the RTC becomes noisy.

Also, given *s*(*t*) and *S*(*t*), comparison of Eq. (14) to Eq. (13) gives

(15) $$r_{N}(t)=\alpha_{BS}(t)\cdot {\left(1-S(t)\right)}^{1/N}=\alpha_{BS}(t)\cdot f_{1}(t)$$

This indicates that the estimating function introduced by Barrett and Stevens can be written in our framework as

(16) $$\alpha_{BS}(t)=\frac{r_{N}(t)}{f_{1}(t)}=N\cdot \frac{r_{1}(t)}{f_{1}(t)}$$

and is therefore always an overestimation of *r*_*N*_(*t*), since Eq. (4) implies that *f*_1_(*t*) ≤ 1 for any value of *t*.

#### 3.2.2 Bias in quantities estimated with the BS formula

We next used these analytical relations to quantify the bias in estimating the RTC with the BS formula if no replacement of released vesicles occurs. To this aim, it is useful to note that Eqs. (2) and (16) imply that, given *r*_1_(*t*), the effect of changing *N* is just a vertical rescaling both for *r*_*N*_(*t*) and for *α*_*BS*_(*t*). Thus, any temporal (“horizontal”) quantity calculated on those two functions, such as FWHM and *τ*, is unchanged if *N* alone is changing. Moreover, since *r*_*N*_(*t*) and *α*_*BS*_(*t*) are rescaled by exactly the same fractional amount, the fractional change of any “vertical” quantity, such as *r*_max_ and *m*, is the same for the two functions. These observations mean that the fractional difference of the quantities depicted in Fig. 1 calculated on *r*_*N*_(*t*) or on *α*_*BS*_(*t*) will not depend on *N* if *r*_1_(*t*) is fixed. It is therefore possible to plot such fractional differences, which quantify the bias of BS estimators with respect to the ones in the binomial (no replacement) model, simply as functions of *P*.

**Fig. 3:**
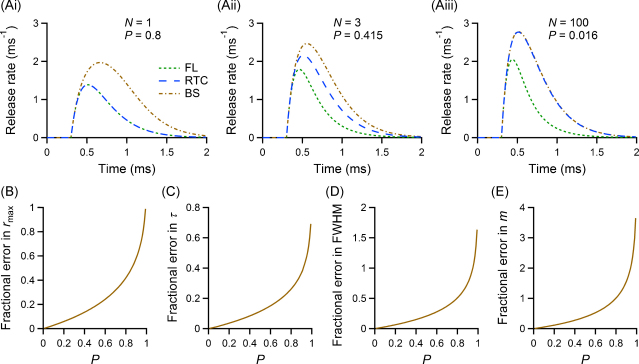
Quantification of the bias in the Barrett and Stevens estimator using analytical derivations. (Ai–Aiii) The first latency curve (FL), release time course (RTC), and the Barrett and Stevens corrected curve (BS) are plotted for 3 synapses, all with a percentage of failures of 20% (*N* and *P* scale as indicated on individual plots). (B–E) The bias in estimating four parameters of the RTC using the BS corrected curve is shown as a function of the vesicular release probability *P*. The parameters *r*_max_, FWHM, *τ*, and *m* are illustrated in Fig. 1. Biases are expressed as fractional error of the quantity calculated from the BS corrected curve with respect to that calculated from the RTC (note the different scales on the Y axes).

We show the results of such analytical analysis in Fig. 3. We compared the time-dependent release rate purely based on the first latency distribution to that obtained using the BS correction and to the actual RTC. All calculations were carried out modelling the release rate with a Gamma distribution with standard deviation of 300 μs, shifted away from the origin (see Section 2), and subsequently applying Eqs. (2), (11) and (14) to obtain the plotted curves. The panels in Fig. 3A show three examples, all for synaptic connections with the same failure probability of 20% as in Fig. 2. Fig. 3Ai shows the case of only one releasable vesicle mentioned in Section 3.1.1. The RTC based on first latencies and the actual RTC overlap, whereas the application of Eq. (14) produces a time course with a difference in half-width (779 μs vs 519 μs), decay time constant (326 μs vs 302 μs) and most notably maximal release rate (1.98 ms^−1^ vs 1.39 ms^−1^) and quantal content (1.61 vs 0.8). For a synaptic connection with few release sites (Fig. 3Aii), the synaptic RTC derived on the basis of the first latencies clearly underestimates the peak release rate, half-width, decay time constant and quantal content (by up to 36% in this example), and applying the Barrett and Stevens correction leads to an overestimate in these parameters by up to 29%. For synapses with a large number of releasable vesicles and a low release probability (Fig. 3Aiii) the BS correction leads to a good estimate of the RTC, whereas the first latency curve leads to an underestimate of parameters by up to 50%. Fig. 3B–E shows the quantification of the differences between the actual RTC and the time course obtained from application of Eq. (14) to the first latency release rate function, using the four quantities introduced in Fig. 1. It is evident that at synaptic connections with low release probabilities and thus lower quantal content (*P* < 0.2) the BS correction method results in reasonable estimates of the RTC. However, for connections with higher release probability an error of up to 50% (*P* ≈ 0.6) and more can be made using the BS correction to deduce the RTC. At the same time, if *N* > 1 the time course obtained on first latencies alone does not give an appropriate estimate either. Thus the true RTC remains unknown.

#### 3.2.3 Regions of validity of the various estimators of the release time course

**Fig. 4:**
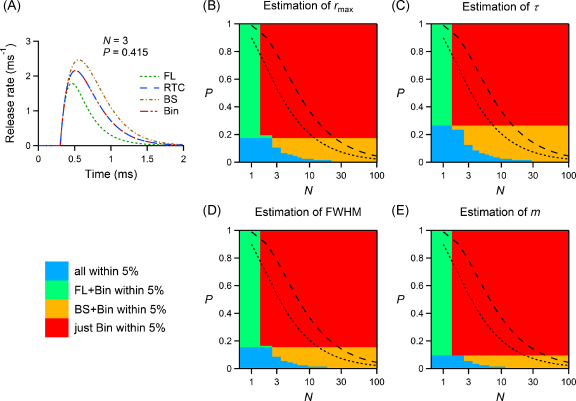
Reliability of release time course estimators over a wide range of parameters. (A) Same plot as in Fig. 3Aii, with the addition of the curve obtained using the binomial correction (Bin), which in this case is the only valid estimator of the RTC. Note that, since analytically computed RTC functions are plotted, Bin = RTC. (B–E) Semi-logarithmic plots showing the regions of the parameter space (indexed with *N* and *P*) where the first latency curve and the BS-corrected curve give an estimation of the RTC parameters with a bias lower than 5%. Note that the BS estimator is never exact, although the plots show the regions where its bias is deemed acceptable. The dotted and dashed lines indicate synapses with percentages of failures of 10% and 1% respectively. The colour legend is displayed under panel (A). (For interpretation of the references to color in this figure legend, the reader is referred to the web version of the article.)

Since the BS correction and the first latency curve alone have been used to estimate the RTC from the first latencies at a variety of synapses, we quantified more explicitly the regions of (*N*, *P*) space where the different approaches produce useful estimates. An example is shown in Fig. 4A, which corresponds to Fig. 3Aii with the addition of the binomial estimate of the RTC (red line), obtained using Eq. (13): the binomial estimate overlays the actual RTC (blue line), whereas in this case both the first latencies and the BS curve clearly lead to erroneous results. In fact, the binomial correction (Bin) was derived under the assumptions of our model and is therefore always correct. To examine how well the first latency and BS curves estimate the RTC, we calculated analytically the values of *N* and *P* where the RTC had a bias smaller than 5%. We then categorized our results to provide an overview of the regions in the (*N*, *P*) space where the various curves give satisfactory estimates (semi-logarithmic plots in Fig. 4B–E). Since estimates of the RTC that are based on a correction of the first latency curve are less reliable when the failure rate is lower, we have also indicated the values of *N* and *P* for synapses with a failure probability of 10% (black dotted line) and 1% (black dashed line). Where a reliable correction of the RTC based on first latencies is possible, there is a range where the binomial formula produces the only acceptable estimates, which corresponds to small synapses (*N* < 10) with intermediate to high vesicular release probability (*P* > 0.2). Fig. 4B–E also shows that for very low release probabilities (*P* < 0.1) the RTC deduced purely based on the first latencies describes the actual RTC well.

### 3.3 Use of the binomial correction under different conditions

We next tested the practical application of the binomial correction, and examined how well it performed under a range of defined conditions and when some of the assumptions underlying its derivation are compromised by non-uniform synaptic properties.

#### 3.3.1 The procedure for estimating the release time course

**Fig. 5:**
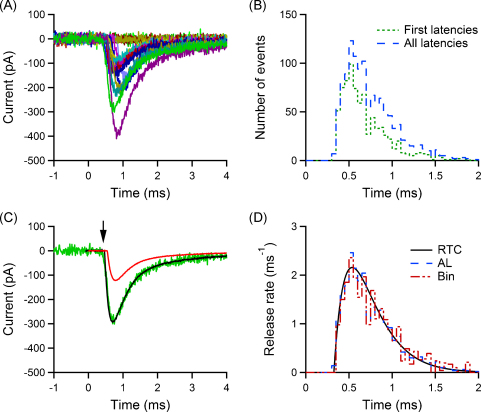
Measuring first latency distributions and using the binomial correction to estimate the release time course. (A) Postsynaptic traces corresponding to 20 sample events at a simulated synapse with parameters equal to that in Fig. 2, and including background noise. (B) Complete histograms of first latencies and all latencies for the simulated experiment, which consists of 1000 stimulations of the same synapse. (C) The quantal postsynaptic response (in red) is superimposed on an individual response (in green, same colour as in the first panel). A scaled and aligned version of the quantal response, obtained with a template match search, is also shown (in black). The algorithm used (see Section 2) then provides an estimate of the first latency (black arrow), regardless the number of quanta present. (D) The estimate of the RTC determined from the measured first latency distribution using the binomial correction is plotted in red (Bin), together with the release rate function for all quanta in this particular simulation (AL; in blue), and the true underlying RTC they were drawn from (in black). (For interpretation of the references to color in this figure legend, the reader is referred to the web version of the article.)

In order to estimate the RTC at a synapse, postsynaptic responses must be recorded and first latencies plotted as a histogram, which can then be corrected with the binomial formula (Eq. (13)). In this section, we test this procedure using stochastic simulations of synaptic release (Section 2) that include quantal variability and background noise (also see Section 3.3.4). A sample of simulated postsynaptic responses for a synapse with parameters identical to that used in Fig. 2 is shown in Fig. 5A, while Fig. 5B depicts the histograms of latencies used in this simulated experiment of 1000 trials. The simulated electrical noise had a standard deviation of 10 pA in this case. Many different methods are available to measure first latencies from the traces; for example, the rising phase of individual responses can be fitted to a line, a parabola or a more complex function, and the intersection of the fit with the baseline can be found, or the point on the fitted response where the 20% of the peak is reached can be used (Feldmeyer et al., 1999; Kraushaar and Jonas, 2000; Sargent et al., 2005). When the signal to noise ratio (SNR), defined as the ratio of the mean peak signal over the standard deviation of noise, is high, it is advisable to use a simple threshold method based on the amplitude of the quantal response. Here, we used a method that can be useful for low SNR as well (see Section 3.3.4). We aligned the mean quantal postsynaptic waveform used in the simulation to each individual response using a widely used template matching algorithm, as detailed in the Methods (Fig. 5C). Experimentally, the quantal response can be obtained separately for the same synapse, for example as an average of miniature responses. This method allowed us to estimate the first latency as the point of rise from the baseline for the aligned template trace (black arrow in Fig. 5C). We then built the first latency histogram using a bin width of 50 μs as in Fig. 2. Once the first latency histogram has been obtained, and has been normalised to a total area of 1 − *F* to give *s*(*t*), *N* needs to be determined (see next Section). The RTC can then be estimated applying Eq. (13). Every bar of the histogram is corrected using the value of *S*(*t*) at the same time bin, which is obtained using Eq. (5). Fig. 5D shows the outcome of the procedure: both the binomial estimate of the RTC and the appropriately scaled version of the histogram including all latencies (AL) matched the RTC underlying this stochastic simulation.

#### 3.3.2 Estimating N

An important caveat of the correction approach presented in Eq. (13) is that although analytically correct, in practice it requires knowledge of *N*, the maximum number of vesicles that can be released by a single action potential. Experimentally, the determination of this quantal parameter can be achieved by applying multiple-probability fluctuation analysis (MPFA, Silver et al., 1998; Silver, 2003), also known as variance-mean analysis (Reid and Clements, 1999; Clements and Silver, 2000). MPFA allows estimation of the quantal parameters of a synapse (in our case *N*, *P* plus the quantal size *Q*) from the relationship between variance and mean amplitude of postsynaptic responses. The method requires collecting at least 100–200 responses at several different release probability conditions. *N*, *P* and *Q* can then be estimated by plotting the variance of the peak amplitude against the mean peak amplitude of each data set, and fitting the result to the appropriate curve using the least squares optimization method (for more details, see Sections 2.1.1 and 3.1.4 in Silver, 2003, and his Fig. 1). This approach has been used to determine *N* at many central synapses (see Section 1.2 in Saviane and Silver, 2006b).

#### 3.3.3 Accuracy of the estimation in different conditions

**Fig. 6:**
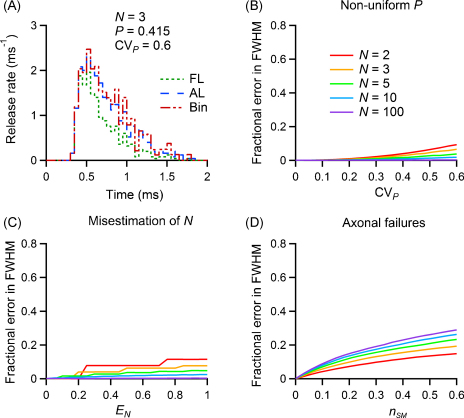
Estimation of the release time course using the binomial correction over a wide range of biologically realistic conditions. (A) Sample histograms for a simulated experiment with 500 stimulations, for a synapse with parameters as in Fig. 2, but having non-uniform *P* with a coefficient of variation (CV_*P*_) of 0.6. (B–D) Results of simulations with large number of trials for synapses with a failure rate of 20% are plotted as the error in estimating the FWHM for various problems, described in detail in Section 3.3.3 (note the different scales on the X axes). (B) Effect of non-uniform *P*, expressed using CV_*P*_. (C) Effect of wrongly estimating the number of vesicles; *E*_*N*_, relative error in *N*. (D) Effect of axonal transmission failures; *n*_SM_, fraction of postsynaptic successes missed.

**Fig. 7:**
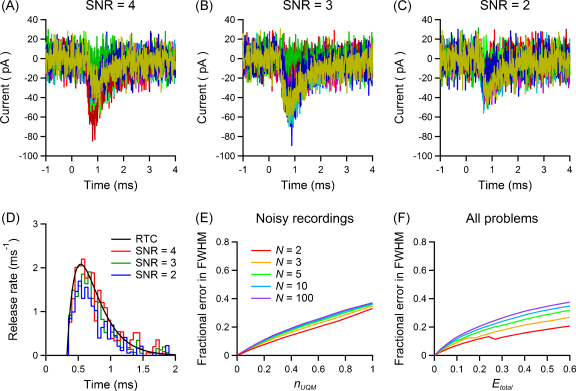
Effect of different levels of noise on the estimation of the release time course. (A–C) Postsynaptic traces of 10 selected events at three simulated synapses with parameters equal to that in Fig. 5A, but with mean peak amplitudes adjusted to obtain SNRs of 4 (A), 3 (B) and 2 (C). (D) RTC estimates obtained with template extraction under the three different SNR conditions using the binomial correction method are plotted in red (traces in A), green (traces in B), blue (traces in C), together with the true underlying RTC they were drawn from (in black). (E) Bias in the estimation of the RTC half-width due to noise, calculated as in Fig. 6; *n*_UQM_, fraction of uniquantal events missed due to the noise level. (F) Bias when the noise is present together with all problems described in Fig. 6, with *E*_*N*_ = CV_*P*_ = *n*_SM_ = *n*_UQM_ = *E*_total_. (For interpretation of the references to color in this figure legend, the reader is referred to the web version of the article.)

Deviations from our model assumptions and other factors can result in errors in the estimation of the RTC when using the binomial correction. We investigated the impact of these with a quantitative analysis of the error in determining the FWHM parameter (Figs. 6 and 7). We used numerical simulations with a large number of trials (see Section 2), for representative cases of synapses having different values of *N* and *P*. For a fixed failure rate of 20%, the two parameters range from *N* = 1 and *P* = 0.8 to *N* = 100 and *P* = 0.016. The values of *N* span a useful range, considering that for *N* = 1 the correction would not modify the first latency curve, while *N* = 100 is already very close to the Poisson limit. In each panel, the absolute value of the error on FWHM is plotted against a variable that quantifies the extent of the particular problem analysed.

Fig. 6A shows sample histograms for the case with non-uniform release probability. The results of the general quantitative analysis are illustrated in the plot shown in Fig. 6B. In this case, *r*_1_(*t*) varies in amplitude (but not in shape) across releasable vesicles, so that each vesicle has a different value of *P*, with an average value $\bar{P}$ and standard deviation *σ*_*P*_. The resulting error is plotted against ${\text{CV}}_{P}=\sigma_{P}/\bar{P}$, ranging from 0 to 60% (a physiologically relevant range: see Murthy et al., 1997; Sargent et al., 2005). For low values of CV_*P*_, the simple binomial correction of the first latency curve gives an excellent estimate of the RTC, with less than 5% error when CV_*P*_ = 30%, and less than 10% error across the whole range analysed.

Fig. 6C shows the effect of a wrong estimation of the number of releasable vesicles. The correction of the first latency curve is applied using a value *N*_L_ = *N*(1 + *E*_*N*_) that is larger than the actual value *N*. The error on FWHM is plotted for each value of the relative error *E*_*N*_, with *N*_L_ being rounded to the closest integer. It is evident that errors on the determination of *N* result in an incorrect estimation of the shape of the RTC, and that these can be as large as 20% for a relative error of 1. In this case, the worst affected parameter would be the quantal content *m* (data not shown).

Another common problem is the presence of failures of axonal transmission in experiments involving stimulation of fibres. Fig. 6D illustrates the resulting error as a function of *n*_SM_, the fraction of postsynaptic successes missed because of this problem, so that for example a value of *n*_SM_ = 0.5 indicates that half the simulated postsynaptic responses were not actually detected as a result of stimulation or transmission failures. The binomial curve was consequently calculated correcting a first latency histogram that was uniformly reduced by *n*_SM_ with respect to the real one, causing an underestimation of the RTC. This causes a biased determination of the half-width with an error of 10% (low *N* values) to 20% (high *N* values) when 30% of the responses are missed. It is therefore advisable to ensure that axon stimulation failures do not contribute significantly, as levels close to 60% would give errors up to 30% in estimating the shape of the RTC.

#### 3.3.4 Estimating the release time course under noisy conditions

The effect of noise was investigated using the procedure in Section 3.3.1 and simulated postsynaptic traces that included quantal variability and noise. It is important to note that, when we compared the histogram of the first latencies measured with the template match search with the histogram of the true first latencies used in that simulation, we detected no significant difference between the two (*p* > 0.05; Kolmogorov–Smirnov test). The SNR was however quite high in this case (>10). We therefore assessed the reliability of the latency measurements for recordings with various levels of noise, by performing a similar analysis on traces with different values of SNR. Fig. 7A–C shows sample traces from simulations with parameters similar to those used for Fig. 2, but gradually decreasing the peak amplitude of responses to obtain a SNR equal to 4 (A), 3 (B) or 2 (C). The corresponding estimations of the RTC of the synapse are shown in Fig. 7D for a SNR = 4 (red), SNR = 3 (green), and SNR = 2 (blue), together with the real underlying RTC (black). In the first case, the test reported no significant difference, and 4% of the smallest (uniquantal) responses were not detected by the template match search; in the other cases the difference was significant (*p* < 0.05), and the percentages of missed uniquantal events were 23% and 33%, respectively. The procedure is therefore robust over a wide range of SNR; however, the analysis shows that the peak release rate and the quantal content are incorrectly estimated when many events are missed.

We also investigated the effect of SNR on RTC. Fig. 7E illustrates the error made in the estimation of the FWHM, using an approach similar to that employed in Section 3.3.3. The error is plotted against the fraction *n*_UQM_ of uniquantal events that were missed because of the high level of noise, and were therefore not included in the first latency histogram. The bias is below 20% for all cases analysed in the paragraph above. However, if the SNR is so low that most uniquantal events are lost in the noise an error in the FWHM of between 30% and 40% can arise, depending on the values of *N* and *P* at the synapse. The last panel of the figure (Fig. 7F) depicts the situation when the problem due to noisy traces is present together with all those described in Section 3.3.3, and all have a comparable relevance, so that *E*_*N*_ = CV_*P*_ = *n*_SM_ = *n*_UQM_ = *E*_total_. Since some of the errors have positive sign (overestimations of the RTC) and some have negative sign (underestimations), in this case they partially compensate each other, giving errors up to 20% for low values of *N* and up to 40% for large *N* in the analysed range. These simulations show that using the binomial correction to estimate RTC from first latencies is robust for synapses with non-uniform quantal parameters and in the presence of noise. However, efforts should be made to minimize errors in *N* and missed events as well as stimulation failures.

#### 3.3.5 Discriminating different release time courses

**Fig. 8:**
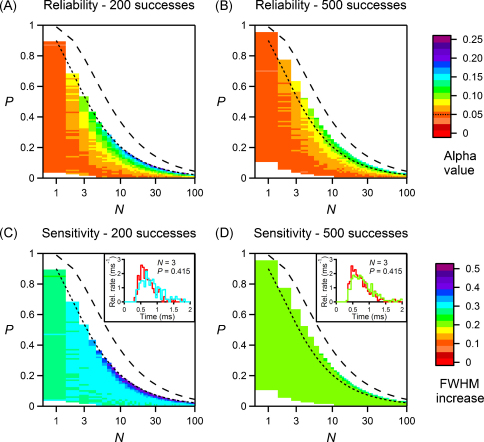
Reliability and sensitivity of estimating changes in release time course from first latencies and binomial correction. Results of simulations with different number of successes are plotted as detailed in Section 2.4.1, just for values of *N* and *P* that guaranteed a failure rate of more than 10% (A and C) or 5% (B and D). Also, simulations were not performed when more than 5000 trials were needed (white). In all panels, the dotted and dashed lines indicate synapses with failure rates of 10% and 1% respectively. (A and B) False positive rate (*α* value) for the comparison of binomial estimates of *τ* and the estimates obtained from the actual latency curve. The colour code is common to panels A and B and is displayed next to panel (B); the dotted line indicates the expected value of 0.05. (C and D) Threshold FWHM increase that guarantees a power of the test of 0.5, when comparing the binomial estimates of *τ* for control synapses and synapses having an RTC with increased FWHM. Insets: sample binomial estimates of the RTC for simulated synapses with the same parameters as in Fig. 2, and with no increase in FWHM (red) or FWHM increased to the threshold value for the represented synapse (blue: 30%, green: 20%). The colour code is common to panels C and D and insets and is displayed next to panel (D). (For interpretation of the references to color in this figure legend, the reader is referred to the web version of the article.)

Changes in the time course of vesicular release can be used to infer information about molecular mechanisms. To determine the resolution with which two RTCs can be discriminated using the binomial corrected first latency distributions, we carried out simulations of the stochastic release process in the absence of background noise. We set up the simulations to mimic the situation when an experimenter compares the RTCs of 10 control synapses (a typical number of experimentally recorded cells) to the RTCs of 10 synapses that may have undergone a perturbation, using the unpaired *t*-test on the estimated values for the parameter *τ* (see Section 2.4.1 for the details). The results of this analysis are illustrated in Fig. 8.

We analysed both the reliability (plots in Fig. 8A and B) and the sensitivity (plots in Fig. 8C and D) of our method for estimating RTC for synapses with *N* ranging from 1 to 100. For each plot, all simulations had the same number of postsynaptic successes, in order to exclude variability due to differences in sampling of the first latency curve. Apart from stochastic variability, the only residual variability is that introduced by applying the binomial correction to the first latency histograms. We excluded from the analysis cases which had a very low quantal content and those with very few failures.

For the reliability analysis illustrated in Fig. 8A and B, the RTC was actually the same in all simulations, but 10 values of *τ* were obtained from actual latency curves, while the remaining 10 were obtained from the binomial corrected first latency distributions. The false positive rate (*α* value) of observing a significant difference between the groups was then estimated over many runs and plotted (see Section 2.4.1 for the details). The *α* value is expected to be 0.05, in the ideal case where the binomial correction did not introduce variability in the estimation. The plots show that in most cases, the false positive rate is not overestimated by more than 0.1, and it is overestimated by less than 0.05 for synapses with at least 10% failures in experiments with 500 successes. This indicates that the additional variability introduced by the Bin correction is quite modest, except when the synaptic failure rate is low.

To test the sensitivity of our method, half of the 20 simulations had an RTC identical to that used in Fig. 2, while the remaining 10 simulations had an RTC with an increased FWHM, and the values of *τ* were estimated from binomial corrected first latency distributions for the control and modified groups. To estimate the minimum change in the RTC that could be detected for an experimentally feasible sample size, we performed a power analysis of the unpaired *t*-test on the control and modified RTC groups. The false negative rate (*β* value) of observing no significant difference was estimated and the power of the test was calculated as 1 − *β*. The whole procedure was repeated for each FWHM increase until the power reached 0.5 (indicating the threshold over which a negative outcome of the test really indicates the absence of an effect with more than random chance probability), and that value of threshold FWHM increase was plotted. Fig. 8C and D show that the threshold value of the FWHM increase is around 30% for experiments with 200 successes, for most synapses with more than one vesicle available, and around 20% for experiments with 500 successes. The insets in Fig. 8C and D illustrate the half-width increases in the RTC of 30% and 20%, respectively.

These stimulations show that estimating the RTC with the binomial corrected first latency distribution is sufficiently sensitive to detect relatively subtle changes in the kinetics of vesicular release, so long as a sufficient number of release events are sampled.

### 3.4 Using first latencies to directly estimate the RTC

**Fig. 9:**
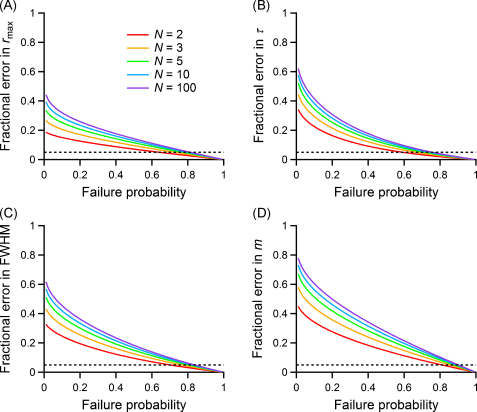
Estimating the release time course directly from the first latency distribution. (A–D) The plots show the bias in estimating the parameters depicted in Fig. 1 when they are obtained from the first latency curve over a range of failure probabilities, calculated using the analytical model. The fractional errors are plotted as a function of the failure probability, for synapses with a number of vesicles ranging from 2 to 100. The black dotted lines indicate an error of 5%.

Fig. 4B–E shows that the RTC is well characterised by uncorrected first latency distribution under low release probability conditions, due to the fact that the probability of overlapping quantal events is low. This can be used in cases when it is difficult to determine *N*. Using analytical calculations, we investigated what error is made when deducing the RTC directly from first latencies as the failure rate of the synapse under investigation varies. Fig. 9 shows the differences in the usual parameters (see Fig. 1) between the actual RTC and the time course obtained using the first latencies of postsynaptic events, for synaptic connections with different numbers of releasable vesicles. Again, the range for *N* is a useful one, considering that under our assumptions the first latencies would directly give an exact estimate of the RTC for *N* = 1. Since knowing the vesicular release probability is as difficult as estimating the number of releasable vesicles correctly (Silver et al., 2003), we plotted the results against the failure probability, which is a function of *N* and *P* that can be easily measured experimentally. The dotted line indicates a 5% difference, which we define as an acceptable deviation from the true RTC. The plots show that the differences in *r*_max_ (Fig. 9A), *τ* (Fig. 9B), FWHM (Fig. 9C), and *m* (Fig. 9D) all become smaller at elevated failure rate. The sensitivity of the four parameters varies, being low for *τ* (a failure probability higher than 60% is always sufficient to estimate it from first latencies with a 10% error), and highest for quantal content. Fig. 9D shows that at a failure probability of over 90% the quantal content can be estimated to 95% accuracy, for all values of *N*. This analysis shows how this method can be a powerful alternative when the failure rate is high. Employing this method in other cases would require increasing the failure rate of the synapse by lowering release probability with manipulations such as low external calcium. This approach assumes that the profile of the RTC does not change with release probability (see Section 4).

## 4 Discussion

The vesicular RTC of a synapse can be directly derived from the latency distribution of the first quantal events to be released in response to presynaptic activation, if the quantal events released later are corrected for. In this study we show that the correction of the first latency histogram proposed by Barrett and Stevens (1972b) cannot be reliably applied to all synapses, particularly not to small central synapses with intermediate to high vesicular release probability. We describe an unbiased correction that allows the RTC to be estimated from the first latency histogram and explain its application over a range of likely experimental conditions using biologically detailed stochastic simulations.

### 4.1 Different models of synaptic release

#### 4.1.1 First latency corrections based on Poisson statistics

The correction originally proposed by Barrett and Stevens (1972b) assumes that vesicular release occurs in a regime of continuous, instantaneous replacement of vesicles, so that it follows Poisson-like statistics (Stevens, 1968). Other authors have used similar formulas, based on Poisson models (see for example: Baldo et al., 1986; Bennett and Kearns, 2000). However, according to the quantum hypothesis (del Castillo and Katz, 1954) the most simple and general way to describe vesicular release is using binomial statistics, provided vesicles are released independently from each other (Kuno, 1964). Binomial release statistics can be approximated by Poisson statistics when the number of releasable vesicles is large and the vesicular release probability is low (del Castillo and Katz, 1954). This is the case at the amphibian NMJ, which was the system studied by Barrett and Stevens (1972a, 1972b) and others performing this kind of analysis (Bennett et al., 1977; Baldo et al., 1986), but it is not true in general. We show that the correction proposed by Barrett and Stevens is instead biased for values of *N*, *P* that are common at central synapses, finding that it can be high for small synapses with intermediate to high *P*.

#### 4.1.2 First latency corrections based on no replacement of released vesicles

The assumption of replacement of vesicles during the release process on a single trial is not likely to be fulfilled at most synaptic connections (see Introduction in Silver, 2003) and it leads to a poor approximation of the release process, particularly in the central nervous system where RRPs can be small. The time window for synchronous phasic release is very brief, ranging from tens or hundreds of microseconds (Sargent et al., 2005; Kanichay and Silver, 2008) to milliseconds (Barrett and Stevens, 1972b). The replenishment of the RRP, on the other hand, is orders of magnitude slower (Rizzoli and Betz, 2005) even in the cases of fast central synapses (Saviane and Silver, 2006a), making it unrealistic to assume any replacement of vesicles during the phasic release process. Consequently, in the model of Barrett and Stevens (1972b) and Stevens (1968) the release function *α*(*t*) is not well defined, as it would actually need to change during the release process, in order to account for the fact that the RRP is changing in size.

We propose a new correction method, which we derive analytically, provided synaptic release obeys the simplest equations implementing the rules of release without vesicle replacement (also see Appendix A). The resulting model shows that in this framework synaptic release is expected to precisely follow simple binomial statistics. It is worth noting that the underlying spontaneous release can still be modelled with a low-probability Poisson or binomial process, but it is in general considered to be negligible during the brief localised periods of fast synchronous synaptic activity (Barrett and Stevens, 1972b).

Detailed descriptions of release are present in the literature already (for review, see Bennett and Kearns, 2000), and many authors have implemented, in various ways, models of transmitter release with no vesicle replacement (Kuno, 1964; Vere-Jones, 1966; Stevens, 1968; Barrett and Stevens, 1972a; Bennett et al., 1977; Bennett and Robinson, 1990; Quastel, 1997). However, the estimation of the RTC from first latencies is never accurately addressed within our simple assumptions (see Section 2.5.1). In some cases, the candidate for the RTC does not actually coincide with the solution of the binomial model (see Stevens, 1968, Eq. (1) in Barrett and Stevens, 1972a, reported also in Bennett et al., 1977). In others, more complicated models with additional parameters are implemented (e.g. constants of mechanisms for explaining the sequestering of unreleased quanta or for the underlying spontaneous release: Bennett et al., 1977; Bennett and Kearns, 2000) or probability density functions normalised to 1 are used instead of our release rate functions, and the estimation of the RTC is not directly dealt with. In particular, none of these cases includes the derivation of a practically useful formula, analogous to Eq. (13).

### 4.2 Suitability of the binomial correction for central synapses

We determined the range of *N* and *P* where the binomial correction gives a significant improvement in the estimation of the RTC of a synapse by assessing the accuracy of the different estimation approaches as the values of *N* and *P* change. For synaptic connections with particularly high quantal content, where no failures are observed over many trials, corrections based on first latencies are not applicable: the RTC cannot be deduced from first latencies, since no information is available about the latencies of quanta released late. Apart from these cases, the binomial correction in Eq. (13) is always reliable for synapses respecting the assumptions in Section 2.5.1, whereas the BS correction fails in large parts of the (*N*, *P*) range and the first latencies alone are often not a good alternative either (Fig. 4). It is clear that many examples of synapses in the central nervous system fall within the region where the binomial correction is the only accurate estimator of the RTC. These include widely studied synapses in the cerebellum, hippocampus and cortex (see among others Dobrunz and Stevens, 1997; Geiger et al., 1997; Kondo and Marty, 1998; Kraushaar and Jonas, 2000; Silver et al., 2003; Sargent et al., 2005; Kanichay and Silver, 2008). Most of these cases also fall in the region of the (*N*, *P*) space where a direct experimental investigation is possible; for the others, in order to carry out experimental investigations and apply this kind of analysis the failure rate would need to be increased, for example in a way similar to that discussed below (see Section 4.4).

The usefulness of the binomial correction was further assessed by characterising both its ability to discriminate different RTCs, and the errors made when using it under conditions such as non-uniform release probability, where the assumptions are compromised. The outcome suggests that the method presented here could be useful for investigating the molecular mechanisms of synaptic release, since changes in the FWHM of the RTCs as small as 20% can be detected for synapses with parameters ranging across the (*N*, *P*) space from first latencies using the binomial formula. Furthermore, the method is robust in that even substantial deviations from our model assumptions do not radically impair the estimation. Misestimation of the number of releasable vesicles and the presence of non-uniform vesicular release probability give errors lower than 20% on the estimated RTC. However, experimenters should be aware that axon stimulation failures can cause more serious errors (30% error with 60% axon failures) at synapses with large *N*. Also, failure to detect many uniquantal events due to very low SNR (lower than 2 in our analysis) can cause errors of up to 40% in the estimation of the RTC.

#### 4.2.1 Use of different functions for the RTC profile

The analytical results are independent of temporal (“horizontal”) rescaling of the RTC. However, the errors made in estimating the release parameters using the BS formula or the first latency curve partially depend on the specific shape of the function underlying the RTC. In all calculations and simulations presented in the Results section, the RTC was described by a Gamma distribution. We also performed a similar analysis using a Gaussian RTC, which resulted in qualitatively similar results (data not shown), although quantitative differences were apparent. Differences arise from the fact that, when using distributions with longer tails (like the Gamma distribution), late first latencies sample the tail of the RTC less well than when using a Gaussian function (shorter tail, decreasing as exp(−*t*^2^)). Other functions can be used, or the width of the release period can change, but it is important to underline that the basic results will not change. Indeed, the estimate for *m* obtained using the binomially corrected first latency curve is independent of the RTC function (*N* · *P*).

### 4.3 Practical considerations for the use of the correction

A general recipe can be derived to guide the experimenter trying to estimate the vesicular RTC at a synapse. Firstly, an important general point needs to be made. This approach assumes that latency distributions arise from a stochastic release process and that each vesicle is triggered at the same time. However, in reality release sites can be dispersed spatially across a dendritic tree. If we analyse a synapse where the jitter within a single site is lower than the dispersion in mean trigger times across sites, it is evident that the RTC will be dominated by axon conduction and not by the release process. For instance, a back-of-the-envelope calculation shows that a synapse with two release sites that are 100 μm apart, where conduction velocity in the axons is 1 m/s, would have latency jitters from conduction alone of around 100 μs, so any asynchrony of the release machinery acting on a time scale smaller than that would get partially masked. This problem arises with most procedures aiming to estimate the RTC from postsynaptic data (included all first latency based methods and most deconvolution methods mentioned in the Introduction). If spatial dispersion of release sites is a significant problem one option is to apply optical quantal analysis, which can be used to measure release at an individual synaptic contact (Oertner et al., 2002).

To measure the first latency distribution the synapse needs to be stimulated at a frequency that is sufficiently low to ensure that the RRP is not depleted, to ensure that *N* vesicles are ready to be released at each stimulation. Adequate frequency ranges will vary according to the speed of vesicle replenishment at the synapse analysed (see the beginning of Section 4.1.2). Also, a sufficiently high number of postsynaptic events need to be collected in order to get a reliable estimate of the latency distributions: the number of stimulations, that includes failures, should be in the order of at least a few hundreds. We used values in the range 500–1500 for most of our simulations, in line with some published experimental work (Barrett and Stevens, 1972b; Baldo et al., 1986; Isaacson and Walmsley, 1995; Taschenberger et al., 2005). The next step is the analysis of postsynaptic recordings for obtaining the histogram of first latencies, as detailed in Section 3.3.1. The main limiting factor for this is the level of noise in the traces; however, for SNRs higher than 4, applying either the template method described or a simple threshold method leads to reliable estimation of the RTC. For higher levels of noise, the plot in Fig. 7E can be a guide to the possible bias in the estimation.

Estimation of the RTC from the first latency histogram requires an estimate of *N*. This can be determined by performing variance-mean analysis (Clements and Silver, 2000; Silver, 2003). Fig. 6C shows the predicted error in the RTC for a misestimation of *N*. It is worth noting that, if just a range is known for *N* (as for the NMJ), it is advisable to apply the binomial correction to the extremes of the range and obtain ranges of values for the RTC parameters. When the upper bound for *N* is high, one of the two extreme corrected curves will be close to that obtained with a Poisson correction under low *P* conditions. At this stage it is important that the experimenter considers the potential biases, including error in estimating *N*, and should proceed if the overall anticipated bias is under a satisfactory level (see Fig. 6B–D and Fig. 7 E and F). If the bias introduced by uncertainty in *N* is unsatisfactory the RTC should be directly estimated from the first latencies under high failure rate conditions, as discussed in section 4.4.

If the goal of the experiment is to determine whether a perturbation caused a change in the RTC, the experimenter can assess the reliability of the result from Fig. 8A and B, by comparing the *α* value for the appropriate values of *N* and *P* with 0.05, since this gives an idea of the false positive rate of the test. Moreover, it may also be instructive to compare the observed fractional change in the RTC with the sensitivity of the approach (Fig. 8C and D), to ensure that any observed significant difference in the RTCs are actually greater than the sensitivity threshold of this approach.

### 4.4 Use of the first latency histogram under low probability conditions

We have also developed an approach that allows the RTC to be estimated without the explicit knowledge of the number of releasable vesicles. This approach relies upon lowering *P* sufficiently to give a low quantal content. But how does the experimenter know when the release probability is low enough to ensure that first latencies yield the release rate without need of further correction? Our theoretical analysis shows that, for failure rates higher than 80%, the probability of overlapping quantal events occurring is so low that the release rate can be directly deduced from the first latency distribution independently of the number of releasable vesicles. This gives an error not bigger than 10% on all parameters used to quantify the RTC. An error of at most 5% is achieved with 90% failure probability, which is in agreement with previous work (Silver, 2003) that estimated the quantal size from the mean amplitude of postsynaptic currents.

Several approaches are available to reduce release probability and increase the number of observed failures of release at a synapse. A standard way is to lower the external Ca^2+^ concentration; alternatively, Cd^2+^ can be included in the external medium (Diamond and Jahr, 1995). The vesicular release probability is thus lowered until the desired failure rate is achieved. These approaches rely on the assumption that the kinetics of the release process is independent of *P*, which has been shown at several synapses but may not be general (Barrett and Stevens, 1972b; Bennett et al., 1977; Datyner and Gage, 1980; Van der Kloot, 1988; Sargent et al., 2005; but see Yoon et al., 2008). An easy way to check if this assumption holds is by comparing the mean time course of the postsynaptic response in the two conditions (however, this can be complicated by probability-dependent receptor desensitization and spillover). Where release kinetics is independent of *P*, the RTC in low release probability conditions is a scaled version of the RTC in physiological conditions. The scaling factor is simply the ratio of the quantal contents in the two conditions, since the quantal content is the integral of the release rate curve. Temporal parameters of the latency histogram, such as the decay time constant and the half-width can then be used as direct estimators of the RTC.

### 4.5 Conclusions

We propose and characterise a new unbiased binomial correction method to obtain the RTC from the first latency distribution of postsynaptic events, which requires the knowledge of the number of releasable vesicles. We use biologically detailed simulations of stochastic release to quantify the sensitivity and reliability and we tests its robustness under conditions of noise, stimulation failure and non-uniform release probability. Based on our analytical considerations and computational modelling results, we discourage the use of the correction developed for the NMJ by Barrett and Stevens to estimate the RTC at small central synapses, since the application of such a correction leads to an error of unknown size, depending on the dimension of the RRP and the release probability. In cases where the number of releasable vesicles cannot be estimated, we show that an attractive alternative to our method is to estimate the RTC directly from the first latency time course under low release probability conditions.

## Appendix A

Here we develop a derivation analogous to the one that led to the BS correction (outlined in Stevens, 1968), and a solution of the difference-differential equation corresponding to his Eqs. (1) and (1a), for the case of a finite number of releasable vesicles. A related approach to this problem is present in the same paper (see Eqs. (10) and (11) in Stevens, 1968), but here we develop the case for no replacement of vesicles (i.e. finite availability), where we explicitly specify the destiny of each individual vesicle separately as for our Eq. (11) in the main text. We use the same definitions and assumptions detailed in Section 2.5.1, and we proceed so that whenever we account for a vesicle in one part of any formula, we make sure that we are not including it anywhere else. In practice this requires that, every time we include a term containing *r*_1_(*t*) to indicate the release of a vesicle *V*_1_, in the same term we divide back the probability that all other vesicles are unreleased by *f*_1_(*t*) (which is the cumulative probability of *V*_1_ not being released), in order to avoid accounting for *V*_1_ twice.

In what follows, we simply rewrite the equations used by Stevens (1968), implementing the finite availability of vesicles by keeping track of each one of them separately in each term. We use the function P[*N*,*k*,t] to indicate the probability of having a situation with exactly (*N* − *k*) unreleased vesicles and *k* released vesicles by a certain time *t*, having started with *N* unreleased vesicles and no released vesicles at time 0.

In this framework, the probability of having the *k*-th release between times *t* and *t* + Δ*t*, when *k* − 1 have occurred up to *t*, can be written as:

$$(N-k+1)\cdot r_{1}(t)\cdot \Delta t\cdot \frac{P[N,k-1,t]}{f_{1}(t)}$$

This is because there are (*N* − *k* + 1) vesicle available, the probability density for a release is *r*_1_(*t*) (so that the probability of a release is *r*_1_(*t*) · *Δt* in the first order in Δ*t*), and we are dividing the probability of having (*k* − 1) vesicles released and (*N* − *k* + 1) unreleased up to time *t* by *f*_1_(*t*), in order to avoid accounting twice for that vesicle released between times *t* and *t* + Δ*t*. Similarly, the probability of having had *k* releases up to time *t* and no more releases between times *t* and *t* + Δ*t* can be written in the first order in Δ*t* as:

$$\left(1-\frac{(N-k)\cdot r_{1}(t)\cdot \Delta t}{f_{1}(t)}\right)\cdot P[N,k,t]$$

This means that using our assumptions to rewrite the starting equation in the description of the Poisson process used by Stevens (1968), the probability of having had *k* releases up to time *t* + Δ*t* can be written in the first order in Δ*t* as:

(A.1) $$P[N,k,t+\Delta t]=\left(1-\frac{(N-k)\cdot r_{1}(t)\cdot \Delta t}{f_{1}(t)}\right)\cdot P[N,k,t]+(N-k+1)\cdot r_{1}(t)\cdot \Delta t\cdot \frac{P[N,k-1,t]}{f_{1}(t)}$$

Note that terms accounting for multiple releases within Δ*t* can be neglected since they are higher order in Δ*t*. Since P[*N*,−1,*t*] = 0 the probability for the case *k* = 0 simplifies to:

(A.2) $$P[N,0,t+\Delta t]=\left(1-\frac{N\cdot r_{1}(t)\cdot \Delta t}{f_{1}(t)}\right)\cdot P[N,0,t]$$

The equations for *k* = 0 (no release) and for *k* > 0 respectively are then:

(A.3a) $$\frac{dP[N,0,t]}{dt}=-N\cdot r_{1}(t)\cdot \frac{P[N,0,t]}{f_{1}(t)}$$

(A.3b) $$\frac{dP[N,k,t]}{dt}=-(N-k)\cdot r_{1}(t)\cdot \frac{P[N,k,t]}{f_{1}(t)}+(N-k+1)\cdot r_{1}(t)\cdot \frac{P[N,k-1,t]}{f_{1}(t)}$$

The first one is a first order linear ODE, and the second one is a difference-differential equation. They can be solved with standard methods, as in Stevens (1968). From Eq. (4) and using the fact that by definition *f*_1_(0) = 1 it is easy to compute the quantity

(A.4) $$L(t)\equiv \int_{0}^{t}\frac{r_{1}(\tau )}{f_{1}(\tau )}d\tau =-\ln(f_{1}(t))$$

The integrating factor for Eqs. (A.3) therefore simplifies to:

(A.5) $$e^{\left(N-k)\cdot L(t\right)}=f_{1}{\left(t\right)}^{-(N-k)}$$

Taken at *k* = 0 this leads to the solution of Eq. (A.3a):

(A.6) $$P[N,0,t]=f_{1}{\left(t\right)}^{N}$$

The initial condition P[*N*,0,0] = 1 was used to choose the appropriate arbitrary constant. Eq. (A.6) can then be substituted into the equation for *k* = 1 and the solution of that can be found using the same procedure. Similar steps can be repeated as for the Poisson case, until it becomes clear that the general solution can be compactly written as the following:

(A.7) $$P[N,k,t]=\left(\begin{array}{c} N\\ k \end{array}\right)\cdot c_{1}{\left(t\right)}^{k}\cdot f_{1}{\left(t\right)}^{N-k}$$

This can be easily verified by substituting Eq. (A.7) into Eqs. (A.3). Thus, under assumptions of release without replacement and uniformity of *r*_1_(*t*) across vesicles, the solution describes a uniform-probability binomial model of release. In fact, remembering that *f*_1_(*t*) = 1 − *c*_1_(*t*), it is clear that the probability function given in Eq. (A.7) describes a binomial model which at every time *t* has binomial parameters *N* and $c_{1}(t)=\int_{0}^{t}r_{1}(\tau )d\tau$. On a time scale much longer than the release process the solution reduces to

(A.8) $$\begin{array}{l} P[N,0,\infty ]={\left(1-P\right)}^{N}\\ P[N,k,\infty ]=\left(\begin{array}{c} N\\ k \end{array}\right)\cdot P^{k}\cdot {\left(1-P\right)}^{N-k} \end{array}$$

which describes a uniform-probability binomial model with binomial parameters *N* and $P=\int_{0}^{\infty }r_{1}(t)dt$. Finally, Eq. (A.3a) and its solution in Eq. (A.6) can be used to obtain Eq. (13) in the same way that Eq. (14) was obtained (Stevens, 1968).

Comparison of this derivation to the one in Stevens (1968) shows that the BS model is the approximation our model converges to in the Poisson limit, when *N*→ ∞ while the quantal content of the release remains constant. In particular, in that limit the formula in Eq. (14) for calculating *α*_*BS*_(*t*) is equivalent to Eq. (13) and yields an unbiased estimate of the RTC.
